# Development of Ti-Nb-Mo-Zr Alloys with Low Modulus and Excellent Plasticity for Biomedical Applications

**DOI:** 10.3390/ma19020325

**Published:** 2026-01-13

**Authors:** Sen Yang, Zhiyuan Jia, Xueyan Song, Junyang He, Xiaoyong Zhang

**Affiliations:** State Key Laboratory of Powder Metallurgy, Central South University, Changsha 410083, China; 233311019@csu.edu.cn (S.Y.); 223301029@csu.edu.cn (Z.J.); 233307005@csu.edu.cn (X.S.); junyanghe@csu.edu.cn (J.H.)

**Keywords:** biomedical titanium alloys, elastic modulus, deformation mechanism, plasticity

## Abstract

Metastable β titanium alloys with low elastic modulus and excellent plasticity represent highly attractive materials for biomedical stent application. Our work shows that Zr plays a crucial role in regulating β stability to significantly reduce the modulus and enhance plasticity. A series of Ti-25Nb-2Mo-xZr (x = 0, 3, 9, 12 wt%) alloys were designed based on the d-electron theory, and the influence of Zr content on the microstructure, mechanical properties, and deformation mechanism were systematically investigated. The results demonstrated that as the Zr content increases, the β phase stability was significantly enhanced. This leads to, first, the suppressed formation of the high modulus α″ phase and ω phase, which results in the decrease in apparent overall elastic modulus. Second, the dominant mode of deformation shifts from martensite dislocation slip (0Zr) to martensitic variant reorientation (3Zr), then to stress-induced martensite transform (SIMT, 9Zr), and finally to a combination of SIMT and deformation twinning (12Zr). Such shifting effectively increases the alloy’s tensile plasticity. Among the series, the Ti-25Nb-2Mo-12Zr alloy exhibited the lowest elastic modulus of 56.3 GPa, together with the highest elongation to failure of 48.2%, demonstrating that the alloy possesses considerable potential for biomedical applications.

## 1. Introduction

Titanium alloys have become widely utilized materials in the biomedical field due to their excellent mechanical properties and superior biocompatibility. However, conventional biomedical titanium alloy, e.g., Ti-6Al-4V and Ti-6Al-7Nb, not only contain biologically toxic elements (Al and V) but also show an elastic modulus markedly higher than that of human bone (10–30 GPa) [1,2,3], which inevitably causes the well-known “stress shielding” effect and the associated risk of implant failure [4,5]. To address this issue, metastable β alloys with a lower elastic modulus have been achieved through the addition of biocompatible elements (such as Nb, Mo, Ta, Zr, and Sn). Examples of such alloys include, Ti-40Nb (wt%) [6], Ti-8Mo (wt%) [7], Ti-70Ta (wt%) [8], Ti-13Nb-13Zr (wt%) [9], Ti-52Zr-14Nb (at%) [10], etc. Although these alloys exhibit reduced elastic modulus (70–90 GPa), they still exceed that of human bone. Further research endeavors for the lower elastic modulus compositional design of titanium alloy are still in urgent demand.

In addition, sufficient plasticity is also critical for the formability of implant materials, especially vascular stent needs to undergo plastic deformation upon deployment [3,11,12,13]. However, titanium alloys with low elastic modulus often exhibit limited ductility and workability, such as the Ti-15Nb-5Zr-4Sn-Fe alloy [14] and the Ti-24Nb-4Zr-8Sn alloy [15]; their elongation is far lower than those of stainless steel and Co-Cr alloy, which typically exceed 40% [16]. This limitation arises from the intrinsic correlation between elastic modulus, plasticity, and β phase stability [17,18,19,20]. When the β phase exhibits low stability, the alloy readily generates large fractions of high-modulus α″ martensite and ω phases. As the β phase stability increases, the microstructure gradually transforms into a single and more stable β phase, giving rise to a characteristic elastic modulus behavior, an initial reduction followed by an increase with further β stabilization [21]. Plasticity is closely associated with the dominant deformation mechanisms. As β phase stability increases, the deformation mode sequentially transitions from transformation-induced plasticity (TRIP) to a combination of twinning-induced plasticity (TWIP) and TRIP, then to TWIP, and finally to dislocation slip [17,22,23,24]. Consequently, plasticity exhibits a trend—initially increasing and then decreasing as the β phase stability increases. Hence, the combination of low modulus and high plasticity can be realized within a narrow window of β stability. Thus, precise control of β phase stability is crucial for developing novel titanium alloys that exhibit both low modulus and excellent plasticity.

Among the biocompatible alloying elements, Nb and Mo have received considerable attention in previous studies due to their strong β-stabilizing effect. Mo, as a high β-stabilizing element, has an intrinsic modulus which is much greater than that of Ti and Nb, and excessive Mo addition may lead to an increase in the β phase stability and modulus of alloys. Interestingly, zirconium (Zr), a non-toxic element with a larger atomic radius than Ti, not only provides solid-solution strengthening but also contributes to β phase stabilization when added together with conventional β-stabilizers, despite typically being considered as a neutral element [25,26,27,28]. For instance, Wang et al. [27] proposed that Zr possesses a theoretical equivalence factor within the Mo equivalent (Mo_eq_) calculation of approximately 0.47, indicating that its β-stabilizing ability is about half that of Mo. According to Pang et al. [25], increasing the Zr content significantly suppresses the formation of the ω phase in the Ti-24Nb (at%). Qian et al. [29] demonstrated that increasing the Zr content caused the dominant deformation mechanism to change from TRIP at 3%Zr to TRIP + TWIP at 6%Zr, and finally to TWIP at 10%Zr in the Ti-12Mo alloy. In addition, in Ti-Nb-Mo-Zr quaternary alloys, Nunes et al. [30] observed that Zr addition tends to reduce young modulus and increase hardness of alloys. Collectively, these findings indicate that Zr plays a critical role in tailoring the stability of β phase and consequently affecting the microstructure features and mechanical properties of alloys. Nevertheless, although several studies have reported on Ti-Nb-Mo-Zr alloys, these investigations have primarily focused on phase constitution. A comprehensive understanding of how Zr regulates β phase stability and how this stability governs deformation mechanisms in Ti–Nb–Mo multi-component alloys remains lacking, warranting further systematic investigation.

Therefore, in this study, the Ti-25Nb-2Mo alloy, following the d-electron theory, was selected as the base composition, and a series of metastable Ti-25Nb-2Mo-xZr (x = 0, 3, 9, 12 wt%) alloys with low elastic modulus and high plasticity were designed with varying Zr content to systematically examine the influence of Zr addition on the mechanical properties and deformation mechanisms. This study provides a theoretical basis and methodological support for the development of next-generation high-performance biomedical titanium alloys for biomedical stent applications.

## 2. Experimental

### 2.1. Design of Ti-Nb-Mo-Zr Alloys

The d-electron theory method represents one of the primary approaches for composition design in the titanium alloy [31,32]. This method involves two parameters: Bo and Md, where Bo refers to the atomic average bond order, and Md refers to the average energy level of d-orbital of the alloying elements. The average $\bar{Bo}$ and $\bar{Md}$ values of the alloy compositions were obtained using the following equations [33,34]:

(1) $$\bar{Bo}=\sum_{i=1}^{n}x_{i}{Bo}_{i}$$

(2) $$\bar{Md}=\sum_{i=1}^{n}x_{i}{Md}_{i}$$

where *x_i_* is the atomic percentage of alloying element *i*, and *Bo_i_* and *Md_i_* refer to the corresponding Bo and Md values of alloying element *i*.

**Figure 1:**
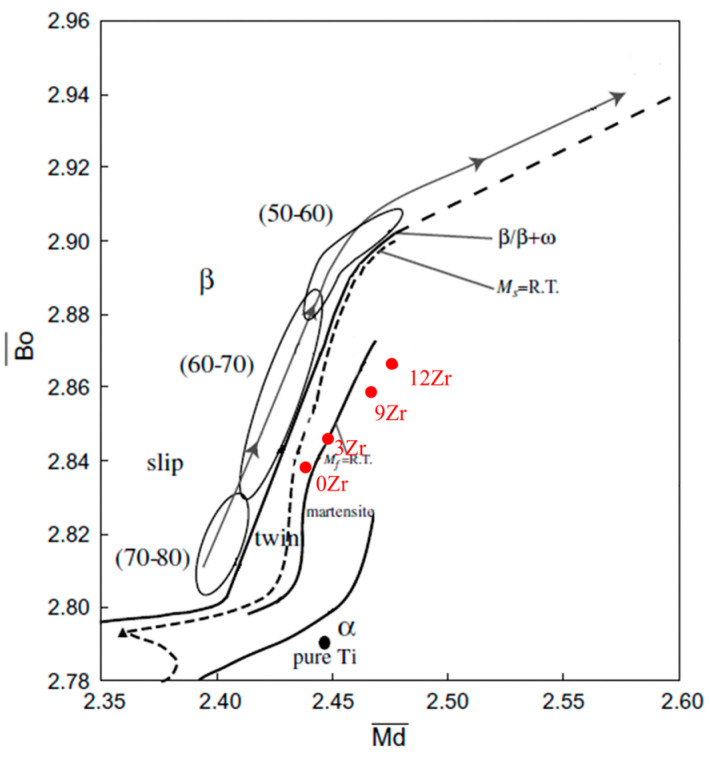
Positions of Ti-25Nb-2Mo-xZr alloys in the $\bar{Bo}-\bar{Md}$ diagram [34].

For β titanium alloys, studies [35] have indicated that higher Bo and Md values are associated with a great potential for achieving lower elastic modulus. Specifically, the low elastic modulus alloys with Bo value in the range of 2.83–2.88 and Md value in the range of 2.43–2.47 [14,34,35,36,37]. Research has demonstrated [37] that the coadding of Zr with β-stabilizing elements promotes simultaneous increases in both Bo and Md values. Based on this, the Ti-25Nb-2Mo alloy, which possesses Bo value of 2.838 and Md value of 2.438, was selected as the base alloy composition. On this basis, the Zr content is added for increasing Bo and Md values to develop alloys with lower elastic modulus. Table 1 lists the Bo and Md values of the designed alloys, while Figure 1 illustrates their corresponding positions within the Bo-Md map.

### 2.2. Methods

Ti-25Nb-2Mo-(0–12) Zr (wt%) alloy ingots (hereinafter substituted by 0Zr, 3Zr, 9Zr, and 12Zr) were fabricated using high-purity Ti (99.9%), Nb (99.5%), Mo (99.5%), and Zr (99.9%) by a vacuum arc melting furnace under a pure Ar atmosphere, and remelted 6 times to ensure a homogeneous composition. The ingots were homogenized at 1100 °C for 2 h in a vacuum tube furnace, followed by water quenching, and subsequently cold-rolled to 1.5 mm sheets at room temperature, with an 80% reduction ratio. Finally, the plates underwent solution treatment at 900 °C for 0.5 h within vacuumed quartz tubes and then were water quenched.

The phase constituents of the alloys were characterized using X-ray diffraction (XRD, PANalytical Empyren, Almelo, The Netherlands) and transmission electron microscopy (TEM, Tecnai F20, Bron, Czech Republic). XRD measurements were performed with Cu Kα radiation over scanning range of 20–80° at a scanning rate of 5°/min. The specimens of the TEM foils were prepared by twin-jet thinning using an electrolyte containing 5% HClO_4_ + 35% C_4_ H_10_O + 60% CH_3_OH at 30 V and −30 °C. Optical microscopy (OM, OLYMPUS, Tokyo, Japan) was employed to observe the microstructure features of the alloys. The samples underwent mechanical polishing and subsequently etched with a solution of 10% HF + 30% HNO_3_ + 60% H_2_O. The deformation mechanisms of the alloys were explored using the electron backscatter diffraction (EBSD, Symmetry S3, Oxford, England, UK). Samples were electrolytically polished for a short period of time (about 30 s) at −30 °C with the same proportion of twin-jet electrolyte to remove residual stresses on the surface caused by mechanical polishing. EBSD data was processed using Aztec Crystal 2.1.2 software.

The mechanical properties of the alloys were obtained from the uniaxial tensile test. Tensile specimens in a dog-bone geometry, featuring a gauge dimension of 20 mm × 4 mm × 1.5 mm, were machined by EDM (RenGuang Numerical Control Equipment Co., Suzhou, China). The tensile experiments were carried out using a CMT4204 testing machine (SUST, Guangzhou, China) under a strain rate of 1 × 10^−3^ s^−1^. An extensometer was employed to obtain accurate measurement of tensile strain. For each alloy composition, three independent specimens were tested to ensure reproducibility. The alloys’ elastic modulus was obtained from the slope of the initial linear segment of the loading stress–strain curve.

## 3. Result

### 3.1. Phase Composition and Microstructures of Ti-25Nb-2Mo-xZr Alloys

**Figure 2:**
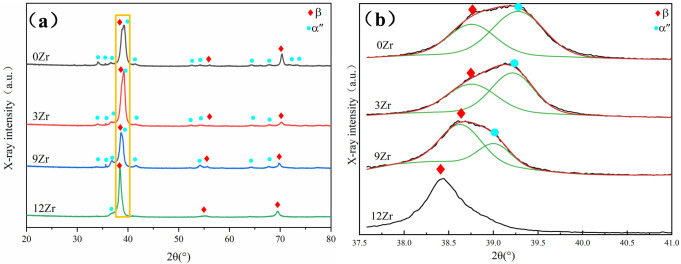
(**a**) XRD patterns of Ti-25Nb-2Mo-xZr alloys; (**b**) magnified section corresponding to the area marked in (**a**).

Figure 2a presents the XRD patterns of the alloys with different Zr contents. All alloys exhibit diffraction peaks of the β and α″ phases. A magnified view of the β(110) diffraction peak is shown in Figure 2b, revealing that the β phase peak progressively shifts to lower angles with increasing Zr content, indicating a change in the lattice parameters of the β phase. The calculated β phase lattice parameters are summarized in Table 2. The lattice parameter gradually increases from 3.2778 Å in the 0Zr alloy to 3.3067 Å in the 12Zr alloy. This shift is attributed to the fact that Zr possesses a larger atomic radius than Ti, which induces lattice expansion. Notably, a strong α″ peak is observed adjacent to the β peak, and its intensity gradually decreases with increasing Zr content. Table 2 also summarizes the lattice parameters of α″ martensite. As the Zr content increases, the lattice parameter a_α″_ shows a gradual increase, while both b_α″_ and c_α″_ exhibit decreasing trends, accompanied by corresponding decreases in the b/a and c/a ratios. These ratio value are commonly employed as indicators of the stability of α″ martensite, and a decrease in their values implies a reduction in α″ martensite stability and gradually transforms to the β phase [38]. Therefore, the suppression of α″ martensite by Zr is primarily associated with its modification of the lattice parameter ratios [39].

**Figure 3:**
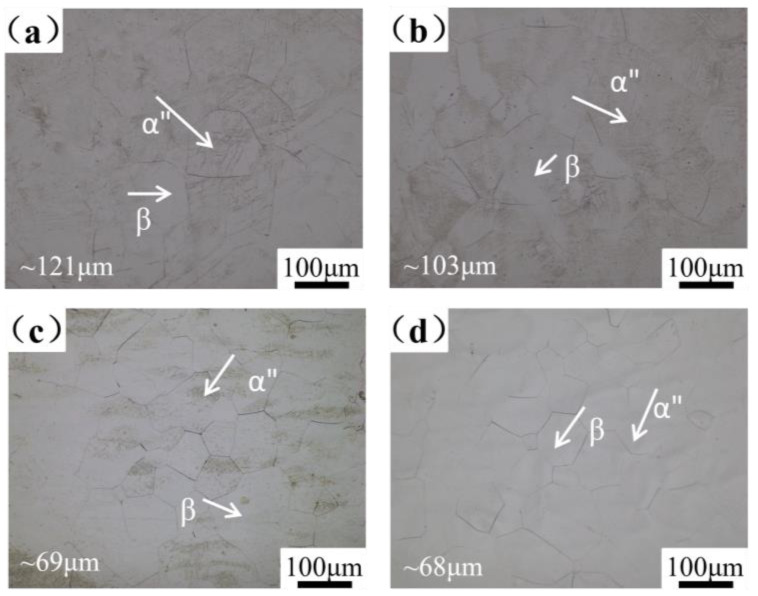
Optical micrographs of alloys. (**a**) 0Zr; (**b**) 3Zr; (**c**) 9Zr; (**d**) 12Zr.

**Figure 4:**
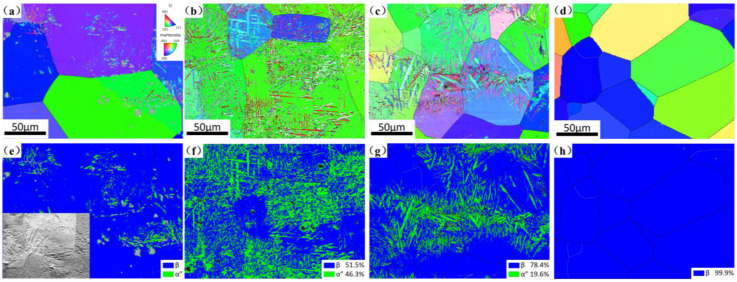
EBSD analysis of the alloys. (**a**–**d**) IPF; (**e**–**h**) phase maps of 0Zr, 3Zr, 9Zr, and 12Zr, respectively.

The optical micrographs of Ti-25Nb-2Mo-xZr alloys are displayed in Figure 3, revealing that all alloys exhibit a β and α″ martensite dual phase microstructure. The grain size of the four alloys is approximately 121 μm, 103 μm, 69 μm, and 68 μm, respectively. Figure 4 presents the EBSD inverse pole figure (IPF) and phase map of alloys with different Zr contents. These α″ martensite exhibit typical characteristic triangular and “V”-shaped morphologies, indicative of a self-accommodating martensitic structure [40]. With increasing Zr content, the fraction of α″ martensite decreases from 46.3% in 3Zr to 19.6% in 9Zr and disappears almost completely in 12Zr. It is worth noting that a relatively high fraction of α″ martensite is present in the 0Zr alloy, but its fine morphology and the limited spatial resolution of EBSD make it difficult to resolve entirely, as seen from the forescatter detector (FSD) illustration.

**Figure 5:**
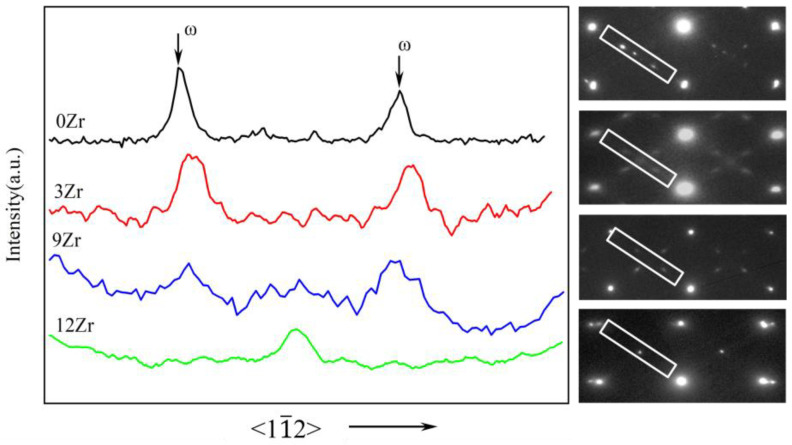
Integrated intensity profiles of selected-area electron diffraction (SAED) patterns along the {112}_β_ direction, normalized to the transmitted beam intensity for 0Zr, 3Zr, 9Zr, and 12Zr alloys.

TEM analysis was employed to confirm the existence of the athermal ω phase. Figure 5 presents the selected-area electron diffraction (SAED) patterns and their corresponding integrated intensity profiles along the {112}_β_ direction for the 0Zr, 3Zr, 9Zr, and 12Zr alloys. Distinct ω phase diffraction spots appear at the 1/3 and 2/3 positions along the {112} direction in the 0Zr, 3Zr, and 9Zr alloys, while no such reflections are detected in the 12Zr alloy. The integrated intensity profiles normalized to the transmitted beam intensity further confirm that the ω phase diffraction peaks progressively weaken with increasing Zr content and eventually disappear in the 12Zr alloy. This indicates that Zr plays a dual suppressive role, inhibiting the formation of both the α″ martensite phase and ω phase.

### 3.2. Mechanical Properties of Ti-25Nb-2Mo-xZr Alloys

**Figure 6:**
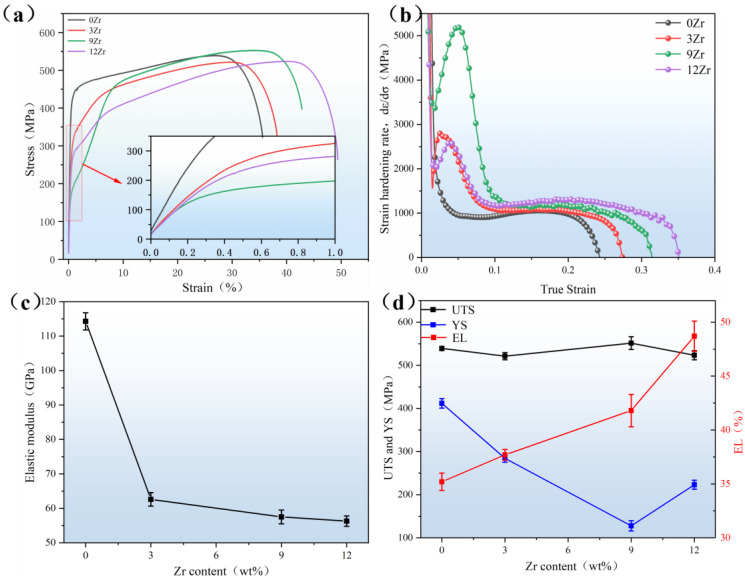
(**a**) Tensile stress–strain curves, (**b**) corresponding strain hardening rate curves, (**c**) elastic modulus, and (**d**) UTS, YS, and EL of Ti-25Nb-2Mo-xZr alloys.

Figure 6a illustrates the tensile stress–strain curves of the xZr alloys at room temperature, and the corresponding mechanical properties obtained from these curves are summarized in Table 3. A single-stage yielding phenomenon is observed in the 0Zr alloy, indicating that plastic deformation occurs prior to α″ martensite reorientation, whereas the 3Zr, 9Zr, and 12Zr alloys show a double-stage yielding phenomenon. The first yield stage is attributed to the α″ martensite variants reorientation and/or the stress-induced martensite transformation (SIMT) due to the coexistence of β and α″ martensite phases in 3Zr, 9Zr, and 12Zr alloy. Figure 6b demonstrates the strain-hardening curves of different alloys, revealing that the 0Zr alloy exhibits a gradual decline in strain hardening, whereas the 3Zr, 9Zr, and 12Zr alloys display a three-stage strain-hardening behavior, similar to that frequently reported in TRIP/TWIP alloys [24,41]. The first stage, characterized by a monotonic decrease, corresponds to the elastic–plastic transition. In the second stage, the strain-hardening rate increases to a maximum value, a behavior associated with the activation of TRIP and/or TWIP mechanisms. In the subsequent stage, the strain-hardening rate declines as deformation-induced microstructural products approach saturation and dislocations accumulate.

Figure 6c demonstrates the relationship between Zr content and elastic modulus. The elastic modulus shows a gradual decreasing trend with increasing Zr content: the 0Zr alloy has a modulus of 114.3 GPa, which gradually decreases to 56.3 GPa when the Zr content reaches 12%. 12Zr alloy demonstrates the lowest elastic modulus among the series. Figure 6d summarizes the relationship between Zr content and the alloys’ ultimate tensile strength (UTS), yield strength (YS), and elongation (EL). The UTS remains relatively stable within the range of 520–550 MPa for (0–12) Zr. The YS exhibits a decreasing-then-increasing trend: it gradually decreases from 411.9 MPa to 127.8 MPa with the increase in Zr content in (0, 3, 9) Zr alloys and then increases to 223.1 MPa in 12Zr. Figure 6d also summarizes the variation in EL with increasing Zr content, showing a clear upward trend. This behavior is attributed to variations in the deformation mechanisms among the alloys, which will be investigated comprehensively in the subsequent sections.

### 3.3. Microstructure of Ti-25Nb-2Mo-xZr Alloy After 10% Strain

**Figure 7:**
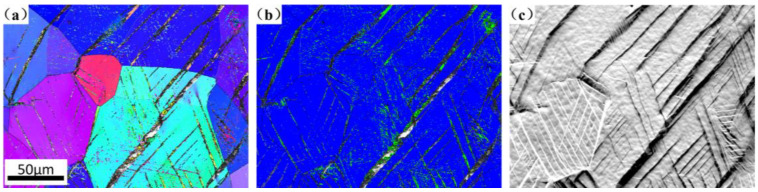
Microstructure of 0Zr alloy after 10% strain. (**a**) IPF; (**b**) phase map; (**c**) FSD map.

As shown in Figure 7, the 0Zr alloy exhibits numerous fine slip traces, together with band-like deformation structures extending across multiple grains after 10% strain, indicating that the dislocation slip is the dominant deformation mode. For alloys containing a high fraction of α″ martensite, the deformation process is typically accommodated by the reorientation of martensite variants, and their stress–strain curve typically exhibit a double-stage yielding characteristic, as reported for alloys such as the Ti-9V-1Fe-4Al alloy [42]. In contrast, the 0Zr alloy, despite also possessing a relatively high martensite content, displays a single-stage yielding phenomenon. This observation suggests that the α″ martensite in the 0Zr alloy is in a relatively stable state. Consequently, the deformation mode of the 0Zr alloy is primarily accommodated by dislocation slip within the α″ martensite, which results in its relatively low elongation.

**Figure 8:**
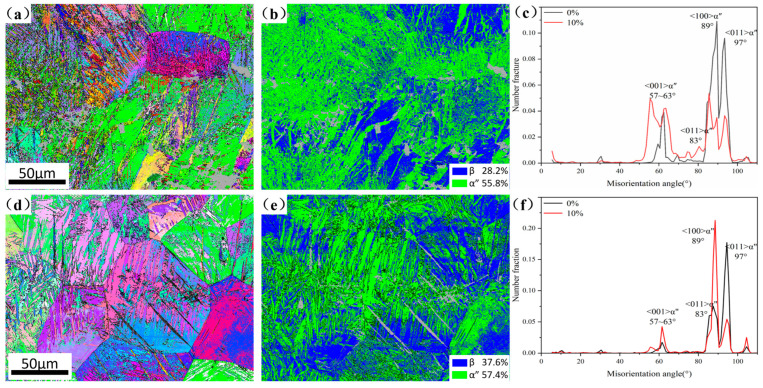
Microstructure of the 3Zr and 9Zr alloys after 10% strain. (**a**–**c**) IPF, phase map, and strain-dependent evolution of the misorientation angle distribution in the 3Zr alloy; (**d**–**f**) IPF, phase map, and strain-dependent evolution of the misorientation angle distribution in the 9Zr alloy.

Figure 8 shows the microstructure of 3Zr and 9Zr alloys after 10% strain. Both alloys initially consisted of β + α″ martensite. As shown in Figure 8b,e, the fraction of α″ martensite increased significantly from 46.3% to 55.8% in 3Zr and from 19.6% to 57.4% in 9Zr. This pronounced increase indicates the occurrence of SIMT, confirming that both alloys exhibit the TRIP effect compared with the 0Zr alloy. In contrast, the extent of martensite formation in the 9Zr alloy is significantly higher than that in the 3Zr alloy, indicating that with increasing Zr content, the phase becomes more prone to deformation-induced instability, thereby activating a stronger TRIP effect. In addition, the orientation relationships corresponding to the different α″ martensite twin types are listed in Table 4 [43], and the misorientation angle distributions before and after deformation are statistically summarized in Figure 8c,f. In the 3Zr alloy, the α″ martensite transforms from {111}α″-type Ι twin and {211}α″-type ΙΙ twin to {130}<$\bar{3}$10>α″ deformation twins, which is a similar phenomenon reported in the Ti-8Mo (wt%) alloy [43] and the Ti-27Nb (at%) alloy [44], while the 9Zr alloy exhibits a transformation from initial twins to {011}α″-compound twin. These results indicate that the initial α″ martensite undergoes variant reorientation during deformation, accompanied by the transformation of the strain-induced residual β phase into α″ martensite. It is noteworthy that the initial martensite content of the 3Zr alloy is higher than 9Zr, suggesting that its deformation behavior is mainly governed by the martensite variant reorientation, whereas the 9Zr alloy mainly deforms through the TRIP mechanism.

**Figure 9:**
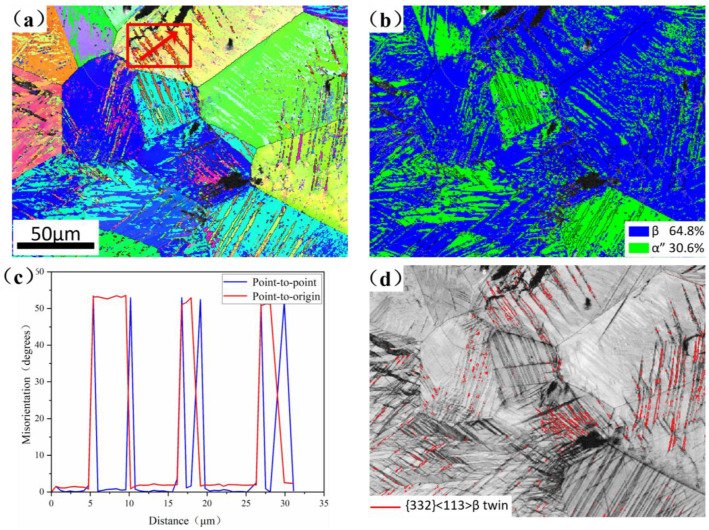
Microstructure of 12Zr alloy after 10% strain. (**a**) IPF and (**b**) phase map; (**c**) misorientation at the red arrow in (**a**); (**d**) {332}<113>_β_ twin boundary.

Figure 9 shows the microstructure of the 12Zr alloy after 10% strain. Figure 9a reveals the formation of numerous α″ martensite lamellae and β twin bands distributed across various grains. The content of martensite increased to 30.6%, indicating that a significant SIMT occurred. As highlighted by the red arrow in Figure 9a, the β twin bands are validated by a misorientation angle of 50.5°, measured and presented in Figure 8c, corresponding to the characteristic {332}<113>_β_ twin. Identification of the {332}_β_ twins is based on the CSL Σ11 criterion, characterized by a roughly 50.5° rotation about the <110> axis [45]. In addition, Figure 9d further quantifies the twinning boundaries, with twin boundaries highlighted in red. Overall, these findings suggest that the deformation in the 12Zr alloy is accommodated through the combined action of TRIP and TWIP mechanisms.

## 4. Discussion

### 4.1. Effect of Zr Content on the Elastic Modulus and Yield Strength of Ti-25Nb-2Mo-xZr Alloys

The mechanical behavior of the Ti-25Nb-2Mo-xZr alloys varies significantly, particularly in elastic modulus and yield strength. As can be seen in Figure 6c, the elastic modulus decreases progressively with increasing Zr content. This is mainly attributed to two factors: (1) interatomic bonding strength and (2) phase construction. As the elastic modulus is inherently determined by interatomic bonding strength, any change in the lattice parameter directly affects the strength of interatomic bonding and consequently influences the elastic modulus [46,47]. Table 2 shows that the lattice parameter of β phase expands with higher Zr content, and corresponding bonding force can be estimated through the formula given below [35]:

(3) $$bonding\;force\propto \frac{\bar{Z_{eff}}\cdot \bar{B_{O}}}{{\bar{M_{d}}}^{2}}$$

where $\bar{Z_{eff}}$, $\bar{B_{O}}$, and $\bar{M_{d}}$ represent the average values of Zeff (effective nuclear charge), Bo, and Md for the alloy. The calculated bonding force values of xZr alloys are 1.769, 1.761, 1.744, and 1.735, respectively. With increasing Zr content, the bonding force exhibits a decreasing trend, indicating that the elastic modulus gradually decreases. Moreover, there are differences in the elastic modulus of various phases. According to the microstructural results in Figure 3 and Figure 5, the fractions of ω and α″ martensite phases gradually decrease as the Zr content increases. Given that both phases exhibit elastic modulus higher than that of the β matrix [7], their reduction in volume fraction leads directly to a reduction in elastic modulus. It should be noted that the elastic modulus of 0Zr and 3Zr decreases sharply, suggesting that the modulus does not vary linearly with bonding force and phase fraction, particularly when the alloy approaches the β/α″ stability boundary. In this range, even a small amount of Zr addition causes significant softening of the β phase, leading to a large reduction in elastic modulus. Therefore, the reduction in elastic modulus with increasing Zr content arises from the combined effects of bonding force, metastable β stability, and phase.

Figure 6d reveals that the yield strength exhibits a decrease followed by an increase with higher Zr content. This trend is influenced by two main factors: (1) the solid solution strengthening effect contributed by the Zr element, which tends to enhance the yield strength, and (2) the martensite start temperature (M_s_) of alloys. Typically, in metastable β titanium alloys, when M_s_ of alloys is above test temperature (room temperature), alloys with a lower M_s_ exhibit lower yield strength. As M_s_ of alloys approaches the room temperature, causing the lattice softening in the β phase and resulting in a minimum in yield strength [48,49]. According to previous studies [39], M_s_ decreases as the b/a and c/a ratios decrease. As shown in Table 2, the b/a and c/a ratio values decrease with increasing Zr, indicating a corresponding decrease in M_s_. In the (0, 3, 9) Zr alloy specimens, the effect of M_s_ has a stronger impact than solid solution strengthening, leading to a declining trend in yield stress. As the Zr content increases to 12%, the influence of solid solution strengthening becomes dominant, resulting in an increase in yield stress.

### 4.2. Effect of Zr Content on the Plasticity, Deformation Mechanisms of Ti-25Nb-2Mo-xZr Alloys

**Figure 10:**
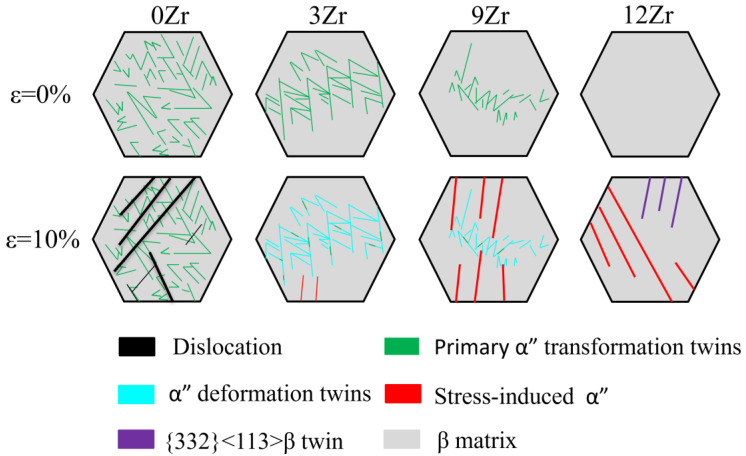
Schematic diagrams illustrating the deformation behavior of Ti-25Nb-2Mo-xZr alloys.

As illustrated in Figure 6d, increasing the Zr content leads to a continuous increase in the elongation of Ti-25Nb-2Mo-xZr alloys. This improvement in ductility is primarily associated with the evolution of deformation mechanisms governed by β phase stability. The corresponding deformation behavior is schematically illustrated in Figure 10. In 0Zr alloy, deformation is mainly accommodated by dislocation slip within α″ martensite, resulting in limited ductility. With increasing Zr content, the β phase becomes more stabilized while the fraction of primary α″ martensite gradually decreases; deformation is progressively dominated by martensite variant reorientation in the 3Zr alloy and TRIP effect in the 9Zr alloy. The activation of the TRIP effect dynamically refines the microstructure by introducing newly formed α″/β interfaces that serve as temporary barriers to dislocation motion. This process produces a dynamic Hall–Petch effect, enhancing strain-hardening capacity of the alloy and postponing the onset of necking, leading to enhanced uniform elongation [17]. When Zr content reaches 12 wt%, the deformation behavior transitions to a combination of TRIP and TWIP mechanisms. Typically, alloys exhibiting both TRIP and TWIP effects tend to achieve superior elongation, followed by alloys dominated by the TRIP effect, and TWIP-dominated alloys typically display lower elongation [18,22,50]. The TWIP/TRIP effect is synergistically coupled to utilize dynamically generated twin/phase boundaries to strongly inhibit dislocation slip, resulting in a dynamic Hall–Petch effect that ensures a optimizes the hardening rate and sustains stable, continuous overall plastic flow while preventing premature cracking [23,51,52]. As a result, the 12Zr alloy exhibits the highest elongation among xZr alloys. This progressive transition demonstrates that Zr addition effectively tunes the stability of the β phase, thereby modulates the relative dominance of various deformation modes, leading to improved overall ductility in metastable β titanium alloys.

### 4.3. Comparison of Mechanical Properties

**Figure 11:**
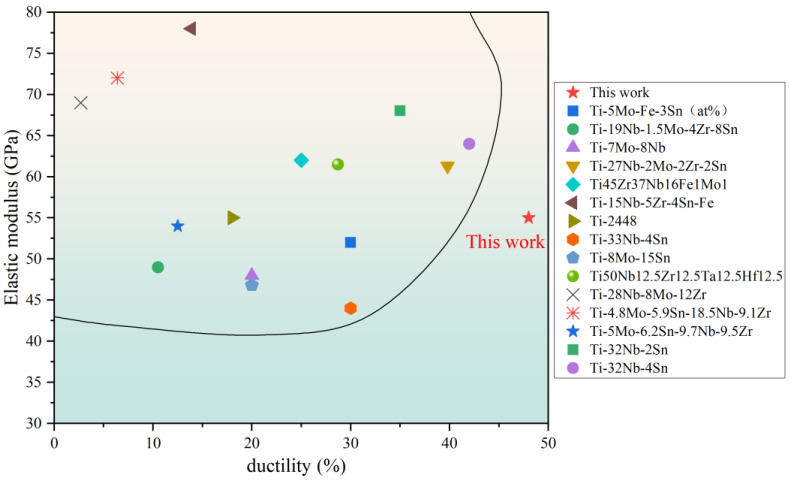
Comparison of mechanical properties between this work and the reported alloys [14,15,49,53,54,55,56,57,58,59,60,61,62].

Figure 11 presents a comparative map of elastic modulus and ductility for previously reported β biomedical titanium alloys. This work’s Ti-25Nb-2Mo-12Zr alloy exhibits a superior combination of low elastic modulus (56.3 GPa) and large ductility (48.2%), demonstrating that the alloy possesses considerable potential for biomedical stent applications.

## 5. Conclusions

In our study, a series of novel Ti-25Nb-2Mo-xZr alloys were designed for potential biomaterials. The influence of varying Zr content on their microstructure evolution, mechanical behavior, and underlying deformation mechanisms were systematically examined. The main conclusions are as follows:

1. The microstructure gradually changes from the ω + α″ + β three-phase of 0Zr to β + minor α″ martensite in the 12Zr as Zr content increases. The addition of Zr content effectively inhibits the formation of the α″ martensite phase and the ω phase.
2. (0–12) Zr alloys exhibit good mechanical properties. With increasing Zr content, the elastic modulus decreases, the yield strength first decreases and then increases, and the plasticity gradually improves. Among these alloys, the 12Zr alloy demonstrates the most superior mechanical properties, including low elastic modulus (56.3 GPa) and high elongation (48.2%), which are expected to be used in future stent biomaterials.
3. As the Zr content increases, the stability of the β phase is progressively enhanced, driving a shift in the dominant deformation mode of the alloy: from dislocation slip within the martensite phase in the 0Zr alloy, to martensitic variant reorientation in the 3Zr alloy, followed by the activation of the TRIP effect in the 9Zr alloy, and finally evolving into a combined TRIP and TWIP effect mechanism in the 12Zr alloy.

## Figures and Tables

**Table 1:** $\bar{Md}$ values of Ti-25Nb-2Mo-xZr alloys.

Alloy (wt%)	$\bar{Bo}$	$\bar{Md}$
Ti-25Nb-2Mo-0Zr	2.838	2.438
Ti-25Nb-2Mo-3Zr	2.845	2.446
Ti-25Nb-2Mo-9Zr	2.858	2.465
Ti-25Nb-2Mo-12Zr	2.865	2.475

**Table 2:** Lattice parameters (Å) of the β and α″ martensite phases in Ti-25Nb-2Mo-xZr alloys.

	β	α″				
		a	b	c	b/a	c/a
0Zr	3.2778	3.0820	5.0497	4.9047	1.6384	1.5914
3Zr	3.2800	3.0858	5.0367	4.8820	1.6322	1.5821
9Zr	3.2945	3.0998	5.0356	4.8740	1.6245	1.5724
12Zr	3.3067					

**Table 3:** The mechanical tensile properties of Ti-25Nb-2Mo-xZr alloys.

	YS/MPa	UTS/MPa	E/GPa	EL/%
0Zr	411.9 ± 11	539.2 ± 4.9	114.3 ± 2.5	35.2 ± 0.8
3Zr	284.4 ± 9.1	521.3 ± 8.5	62.9 ± 1.9	37.7 ± 0.5
9Zr	127.8 ± 11.8	551.5 ± 14.9	57.5 ± 2	41.8 ± 1.5
12Zr	223.1 ± 10.5	523.8 ± 10.7	56.3 ± 1.5	48.2 ± 1.4

**Table 4:** Misorientation angle and corresponding rotation axis of transformation and deformation twins in α” martensite [43].

	Twining Type	Misorientation Angle	Rotation Axis
Transformation twin	{111}α″-type Ι twin	97°	[011] α″
	{211}α″-type ΙΙ twin	83°	[011] α″
	{011}α″-compound twin	89°	[100] α″
Deformation twin	{110}<1$\overset{-}{1}$0>α″ twin	62°	[001] α″
	{130}<$\bar{3}$10>α″ twin	58°	[001] α″

## Data Availability

The original contributions presented in this study are included in the article. Further inquiries can be directed to the corresponding author.
